# Deep Brain Stimulation Does Not Modulate Auditory-Motor Integration of Speech in Parkinson's Disease

**DOI:** 10.3389/fneur.2020.00655

**Published:** 2020-07-10

**Authors:** Bahne H. Bahners, Esther Florin, Julian Rohrhuber, Holger Krause, Jan Hirschmann, Ruben van de Vijver, Alfons Schnitzler, Markus Butz

**Affiliations:** ^1^Institute of Clinical Neuroscience and Medical Psychology, Medical Faculty, Heinrich Heine University Düsseldorf, Düsseldorf, Germany; ^2^Center for Movement Disorders and Neuromodulation, Department of Neurology, Medical Faculty, Heinrich Heine University Düsseldorf, Düsseldorf, Germany; ^3^Institute of Linguistics and Information Science, Heinrich Heine University Düsseldorf, Düsseldorf, Germany; ^4^Department of Neurology, Center for Movement Disorders and Neuromodulation, Medical Faculty, University Hospital Düsseldorf, Düsseldorf, Germany

**Keywords:** auditory feedback, subthalamic nucleus, auditory cortex, event related fields, magnetoencephalography (MEG), artifacts

## Abstract

Deep brain stimulation (DBS) has significant effects on motor symptoms in Parkinson's disease (PD), but existing studies on the effect of DBS on speech are rather inconclusive. It is assumed that deficits in auditory-motor integration strongly contribute to Parkinsonian speech pathology. The aim of the present study was to assess whether subthalamic DBS can modulate these deficits. Twenty PD patients (15 male, 5 female; 62.4 ± 6.7 years) with subthalamic DBS were exposed to pitch-shifted acoustic feedback during vowel vocalization and subsequent listening. Voice and brain activity were measured ON and OFF stimulation using magnetoencephalography (MEG). Vocal responses and auditory evoked responses time locked to the onset of pitch-shifted feedback were examined. A positive correlation between vocal response magnitude and pitch variability was observed for both, stimulation ON and OFF (ON: *r* = 0.722, *p* < 0.001, OFF: *r* = 0.746, *p* < 0.001). However, no differences of vocal responses to pitch-shifted feedback between the stimulation conditions were found [*t*_(19)_ = −0.245, *p* = 0.809, *d* = −0.055]. P200m amplitudes of event related fields (ERF) of left and right auditory cortex (AC) and superior temporal gyrus (STG) were significantly larger during listening [left AC P200m: *F*_(1, 19)_ = 10.241, *p* = 0.005, *f* = 0.734; right STG P200m: *F*_(1, 19)_ = 8.393, *p* = 0.009, *f* = 0.664]. Subthalamic DBS appears to have no substantial effect on vocal compensations, although it has been suggested that auditory-motor integration deficits contribute to higher vocal response magnitudes in pitch perturbation experiments with PD patients. Thus, DBS seems to be limited in modulating auditory-motor integration of speech in PD.

## Introduction

Deep brain stimulation (DBS) is known to have strong beneficial effects on motor symptoms in Parkinson's disease (PD) (1, 2). However, research on the effects of DBS on speech is rather inconclusive and the effects seem to critically depend on unidentified individual factors (3, 4). Untreated, up to 90% of PD patients develop severe speech or swallowing difficulties in the later course of their disease (5). In particular, reduced voice volume (hypophonia) and monopitch (hypoprosodia) are typically part of speech characteristics in PD (6).

Sensorimotor deficits are thought to contribute to speech symptoms in PD like hypophonia and hypoprosodia (7). Many PD patients tend to overestimate their own voices' volume and therefore, reduce their output volume (8, 9). When changing auditory feedback in loudness, PD patients compensate the amplitude of their voices substantially stronger than healthy individuals (10). The reduced ability in modulation of pitch represents a significant component of dysprosody and may be at least partly explained by sensorimotor deficits in auditory-motor integration as well (6, 11).

To scrutinize the neural mechanisms underlying auditory-motor integration of speech, pitch perturbation experiments were used in earlier studies (10, 12, 13). In these experiments, auditory feedback is artificially pitch-shifted in real-time and a vocal compensation to these changes is provoked. PD patients compensated stronger to pitch-shifted feedback than healthy individuals and their vocal response magnitudes correlated with the pitch variability of unaltered vowel vocalizations (12, 13). This suggests, that PD patients rely more on auditory feedback during speech production than healthy individuals, reflecting deficits of auditory-motor integration of speech in PD (13). Interestingly, the amplitude of the P200 event-related potential was larger for patients in this experiment. These P200 responses demonstrated a left-lateralized cortical activation pattern, including superior and inferior frontal gyrus (SFG/IFG), premotor cortex (PMC), inferior parietal lobule (IPL), and superior temporal gyrus (STG) (13).

Recent work suggested that the subthalamic nucleus (STN) is also involved in speech production. Increased activity in the gamma band in the STN was shown before speech onset and during articulation, similar to the sensorimotor cortex (14). In fact, DBS of the STN can alleviate specific speech symptoms like hypophonia and voice tremor (15). A trend toward increasing loudness and pitch variability of fluent speech could also be observed (3). Yet, in some patients DBS can also lead to speech deterioration based on perceptual ratings, acoustical measures of verbal fluency, as well as self-reported speech difficulties (16, 17).

In the present study, we aimed at revealing how DBS of the STN influences the auditory-motor integration of speech. To this end, we studied behavioral and neurophysiological responses in a pitch perturbation experiment using magnetoencephalography (MEG). Due to deficits in auditory-motor integration, we expected PD patients to overestimate auditory feedback changes and therefore, to compensate strongly to pitch-shifted feedback (12, 13), especially when DBS is turned OFF. If increased pitch variability during vocalizations is associated with deficits in auditory-motor integration (13), vocal response magnitudes will correlate with pitch variability. Previous work already demonstrated ameliorating tendencies of DBS on the acoustic measure of pitch variability, representing an improved modulation of pitch in fluent speech (3, 4). Therefore, we expected that turning DBS ON would attenuate vocal response magnitudes, i.e., diminish vocal compensations toward similar magnitudes as in healthy individuals (4, 13). Additionally, we hypothesized that P200m amplitudes would be reduced accordingly when the stimulation is turned ON as P200 amplitudes have been suggested to represent the neural correlate of auditory-motor integration deficits in speech perturbation experiments (13). Finally, we expected task specific differences of ERF amplitudes, that have been described earlier in pitch perturbation experiments (18, 19).

## Patients and Methods

### Patients

Twenty German speaking PD patients (15 male, 5 female; 62.4 ± 6.7 years) were recruited during their annual DBS control visit at the Center for Movement Disorders and Neuromodulation at the University Hospital Düsseldorf. The mean disease duration was 9.6 ± 4.4 years. All patients were right-handed as assessed by the Edinburgh Handedness Test (20). The patients were implanted with a DBS system targeting the STN 24.9 ± 21.3 months prior to testing and had a significant therapeutic effect with their respective clinically used monopolar stimulation settings regarding motor scores of the unified Parkinson's Disease rating scale (UPDRS III) (ON: 15 ± 6, *p* < 0.001 vs. OFF: 28 ± 12). UPDRS scores provided in Table 1 have been rated with monopolar stimulation settings as part of the clinical evaluation of the DBS control visit. To minimize DBS artifacts, stimulation was switched from monopolar to bipolar for MEG recordings (Table 1). These bipolar settings were installed and evaluated by a physician trained in DBS programming. Stimulation amplitudes were raised to values below individual side effect thresholds, if the stimulation did not suppress motor symptoms sufficiently. To achieve an equivalent clinical effect and a similar volume of tissue activated (VTA) of bipolar stimulation compared to monopolar stimulation an amplitude increase of about 30% has been suggested earlier (21–23). During MEG recordings patients were in their medication ON state. The levodopa equivalent daily doses (LEDD) can be found in Table 1. The study was approved by the local ethics committee (study number: 6211) and performed in accordance with the Declaration of Helsinki (24). All patients gave their prior written informed consent.

**Table 1:** DBS settings, contacts (L, left; R, right), age group, length of DBS treatment, UPDRS III scores, and levodopa equivalent daily dose (LEDD) of all patients.

**Patient**	**Age group**	**DBS system**	**Cont L**	**Cont R**	**Parameters L**	**Parameters R**	**DBS** **(months)**	**UPDRS** **OFF**	**UPDRS** **ON**	**LEDD** **(mg)**
1	61–65	Abbott Infinity	2a–/4+	10b–/12+	1.7 mA, 60 μs, 130 Hz	1.3 mA, 60 μs, 130 Hz	15	47	19	360
2	56–60	Abbott Infinity	OFF	11a–/9+	OFF	3.4 mA, 60 μs, 130 Hz	54	27	5	250
3	56–60	Boston Scientific	4–/8+	12–/16+	2.8 mA, 60 μs, 130 Hz	3.3 mA, 60 μs, 130 Hz	62	51	29	400
4	56–60	Abbott Infinity	11–/12+	3–/4+	1.0 mA, 60 μs, 130 Hz	0.5 mA, 60 μs, 130 Hz	3	14	11	300
5	50–55	Boston Scientific	4–/5+	12–/13+	1.1 mA, 60 μs, 130 Hz	4.0 mA, 60 μs, 130 Hz	34	25	19	400
6	66–70	Abbott Infinity	2a–/4+	10a–/12+	2.2 mA, 50 μs, 130 Hz	1.8 mA, 50 μs, 130 Hz	5	27	12	925
7	50–55	Abbott Infinity	10bc–/12+	2bc–/4+	4.0 mA, 60 μs, 190 Hz	0.5 mA, 60 μs, 190 Hz	5	16	8	400
8	66–70	Boston Scientific	2–/8+	10–/16+	1.8 mA, 40 μs, 119 Hz	1.7 mA, 40 μs, 119 Hz	60	24	22	898
9	66–70	Abbott Infinity	1–/4+	10a–/12+	1.5 mA, 60 μs, 130 Hz	1.3 mA, 60 μs, 130 Hz	19	17	15	433
10	76–80	Abbott Infinity	2c–/4+	10bc–,11b–/12+	0.8 mA, 60 μs, 130 Hz	4.2 mA, 60 μs, 130 Hz	24	21	7	515
11	56–60	Abbott Infinity	2c–/4+	10c–/12+	2.3 mA, 60 μs, 130 Hz	1.5 mA, 60 μs, 130 Hz	18	9	9	300
12	50–55	Boston Scientific	4,5–/8+	11,12–/16+	2.4 mA, 40 μs, 130 Hz	3.1 mA, 40 μs, 130 Hz	62	22	10	859
13	66–70	Abbott Infinity	2a–/4+	10a–/12+	2.7 mA, 40 μs, 130 Hz	2.1 mA, 40 μs, 130 Hz	21	22	16	1,743
14	71–75	Abbott Infinity	1–/4+	9–/12+	1.5 mA, 50 μs, 130 Hz	1.5 mA, 50 μs, 130 Hz	5	54	19	1,383
15	50–55	Boston Scientific	2, 3–/8+	12–/16+	2.0 mA, 60 μs, 130 Hz	0.9 mA, 60 μs, 130 Hz	51	17	6	810
16	56–60	Abbott Infinity	12–/9+	4–/1+	4.0 mA, 50 μs, 130 Hz	3.4 mA, 50 μs, 130 Hz	37	32	20	740
17	61–65	Boston Scientific	2–, 4–/8+	10–, 12–/16+	3.2 mA, 60 μs, 130 Hz	3.2 mA, 60 μs, 130 Hz	12	32	17	708
18	66–70	Abbott Infinity	3–/4+	11–/12+	3.5 mA, 60 μs, 130 Hz	3.9 mA, 60 μs, 130 Hz	4	39	21	300
19	66–70	Abbott Infinity	2–/4+	10ab–/12+	2.0 mA, 60 μs, 130 Hz	2.5 mA, 60 μs, 130 Hz	3	28	25	710
20	56–60	Abbott Infinity	10–/12+	2–/4+	3.0 mA, 50 μs, 130 Hz	3.0 mA, 50 μs, 130 Hz	4	33	13	675
Mean	62.4				2.12 mA	2.27 mA	24.9	28	15	655.54
*SD*	6.7				1.08 mA	1.22 mA	21.3	12	6	385.11

*Mean age, stimulation amplitudes, UPDRS scores, and LEDD with standard deviations (SD) in the bottom row*.

### Procedure

After turning stimulation OFF for ≥30 min, patients were comfortably seated in the MEG scanner, asked to vocalize the German vowel “E” [e] (vocal task) and instructed to hold their tone irrespective of changes in the feedback. Each block consisted of 30 vocalizations. A visible count-down from 3 to 1, lasting 3 s in total, was displayed on a screen to prepare patients for vocalization. Afterwards, a blue circle containing the letter “E” appeared, indicating the beginning of the first vocalization period. The circle disappeared clockwise within 6 s. A white screen indicated a pause including the count-down for the next vocalization period. These pauses increased from 5 to 9 s to prevent vocal fatigue toward the end of the experiment. During each vocalization period, patients' voice was pitch-shifted downwards 200 cents (two semitones) for 200 ms up to 6 times, which resulted in a maximum number of 161 trials (mean OFF = 125.6, 95%-CI [102.1, 149.0]; mean ON = 126.5, 95%-CI [107.3, 145.6]; Supplementary Table 1). Thereby, the number of pitch-shifts depended on the consistency and duration of vocalizations. Pitch shifting happened randomly at 500–1,000 ms after speech onset with random inter-stimulus intervals (ISI) of 700–900 ms between subsequent pitch changes to avoid habituation. The mean ISI was 0.798 s (95%-CI [0.795, 0.800], max: 0.905 s; min: 0.699 s). Thus, the mean stimulus delivery rate (SDR) estimates to 1.25 Hz (max: 1.10 Hz; min: 1.43 Hz). The altered as well as the unaltered vocalizations were recorded. Afterwards, the recording—including the pitch-shifted sequences—was played back to the patient (listen task). The entire experiment was repeated after stimulation was turned ON again for ≥30 min. The time of each pitch shifting onset was saved in a separate audio file. Subsequently, this information was used to extract the time locked pitch response contours of each trial offline.

### Apparatus

An optical microphone (Sennheiser MO 2000, Wedemark, Germany) was installed at a distance of 5 cm to the patients' mouth. After the signal was processed on the computer's built-in audio interface (SoundMX integrated Digital HD, Intel Corporation©, 64 bits; 33 MHz), a pitch-shifted signal was played back to the participant through insert earphones (ER-1, Etymotic Research Inc., Illinois, USA) via a mixing console (Behringer© XENYX 502 PA). The built-in audio interface had a hardware delay of 5.4 ms and recordings were sampled at 96 kHz. The audio system was calibrated, so that the feedback channel was more than 10 dB louder than the input channel of patients' voice (18). A dummy head microphone (Neumann KU100, Berlin, Germany), typically used for binaural audio recordings, was utilized to calibrate the system. Additionally, a visual presentation with the experimental instructions was executed on another computer independent from sound-processing and displayed using a rear projection system.

### Pitch Shifting

The experiment required a small change in pitch of a voice signal in real-time without changing its intraspectral relations. Not changing these relations allows us to largely preserve the voice signal's natural sound, thereby avoiding possible dynamic artifacts and assuring that the patients still recognize their own voice. To this end, we employed a novel custom-made setup for real-time speech perturbation experiments (25). The speech signal was recorded online into a 4 s buffer pre-allocated to allow for the maximal duration of the pitch shift. During the pitch shifting, its play-back rate was reduced to 0.891 ≈2^−200/1, 200^ using cubic interpolation, effectively lowering the signal's pitch by 200 cents. This modified signal replaced the live signal for 200 ms. It was cross-faded back to the live signal over a period of 100 ms. This simple method is easily replicable and adjustable in SuperCollider (26) using our source code, which is publicly available online under the GNU general public license, version 3 (https://github.com/musikinformatik/pspeech).

### MEG Acquisition

During the experiment, neuromagnetic activity was measured using MEG (Elekta Oy, Helsinki, Finland). Prior to this, patients' head shape and head position indicator coils (HPI) were digitized by means of a 3D-digitizer (Fastrak Digitizer, Polhemus©, Vermont, USA). Eye movements (EOG) and heart activity (ECG) were monitored throughout the measurements. Additionally, the audio signal played-back to the patients was taken from the 2-track output of the mixing console to record it synchronously with the MEG data via one of the miscellaneous channels of the MEG system. Larynx accelerations were acquired the same way. These were measured by the use of a MEG compatible accelerometer to monitor vocalization periods independently from sound (27). To mark pitch shifting events in the MEG data, we sent transistor-transistor logic (TTL) pulses via a parallel port from the experimental computer to the MEG acquisition computer. The pulses were generated with a simple shell script activated by SuperCollider. Each vocalization on- and offset as well as pitch shifting on- and offset was encoded as a specific TTL trigger pulse. Precision of TTL pulses was adjusted with the help of the miscellaneous channels of larynx acceleration monitoring and the original audio signal with a hardware induced jitter of ±1 ms. The combined audio delay of hardware and software components was measured with a SuperCollider script resulting in 9 ms delay. Taking the microphone as well as the ear insert headphones into account, the total delay amounted to 10 ms.

### Vocal Response Analysis

To analyze vocal compensating responses to pitch-shifted feedback, we extracted the individual pitch contours of every patient's recording in PRAAT, a free computer software package for speech analysis in phonetics (28). The pitch contours were transferred to the cent scale (4). We then extracted the responses time locked to the pitch shifting onset, using voice fundamental frequency (f0) values 100 ms before and 500 ms after the onset. By rejecting trials with negative response magnitude values (following responses), we assured that only opposing, i.e., compensating responses, were considered for the response analysis (12). The mean downward response magnitudes were: OFF: 6.05 cents and ON: 8.77 cents. In average 3.9% of trials in OFF and 3.8% of trials in ON were rejected due to following (downward) responses. We also excluded trials that were omitted in MEG preprocessing, so that the number of averaged trials was the same for vocal response and MEG analysis (Supplementary Table 1). We calculated the response magnitude by subtracting the mean baseline f0 (100 ms before pitch shifting onset) from the maximum f0 value in a time window of 100–300 ms after the pitch shifting (13). We made this calculation for each trial and averaged the response magnitudes. The standard deviation of f0 in the baseline period (see above) was calculated as a measure for pitch variability. Also, voice intensity and voice jitter (f0 cycle-to-cycle perturbation) were extracted from the vocalization sequences before pitch shifts to assess overall voice quality. Voice jitter was calculated as the average absolute difference between voice f0 of consecutive cycles (4). After extraction, all further processing was conducted in MATLAB (2018b, MathWorks Inc.).

### MEG Data Analysis

MEG data was sampled at 1,000 Hz with a high-pass filter of 0.1 Hz and a low-pass filter of 330 Hz. The analyses were restricted to the 204 gradiometers of the MEG system. Data analysis was performed with Brainstorm (29), a documented and freely available toolbox for the analysis of brain signals (http://neuroimage.usc.edu/brainstorm).

**Figure 1:**
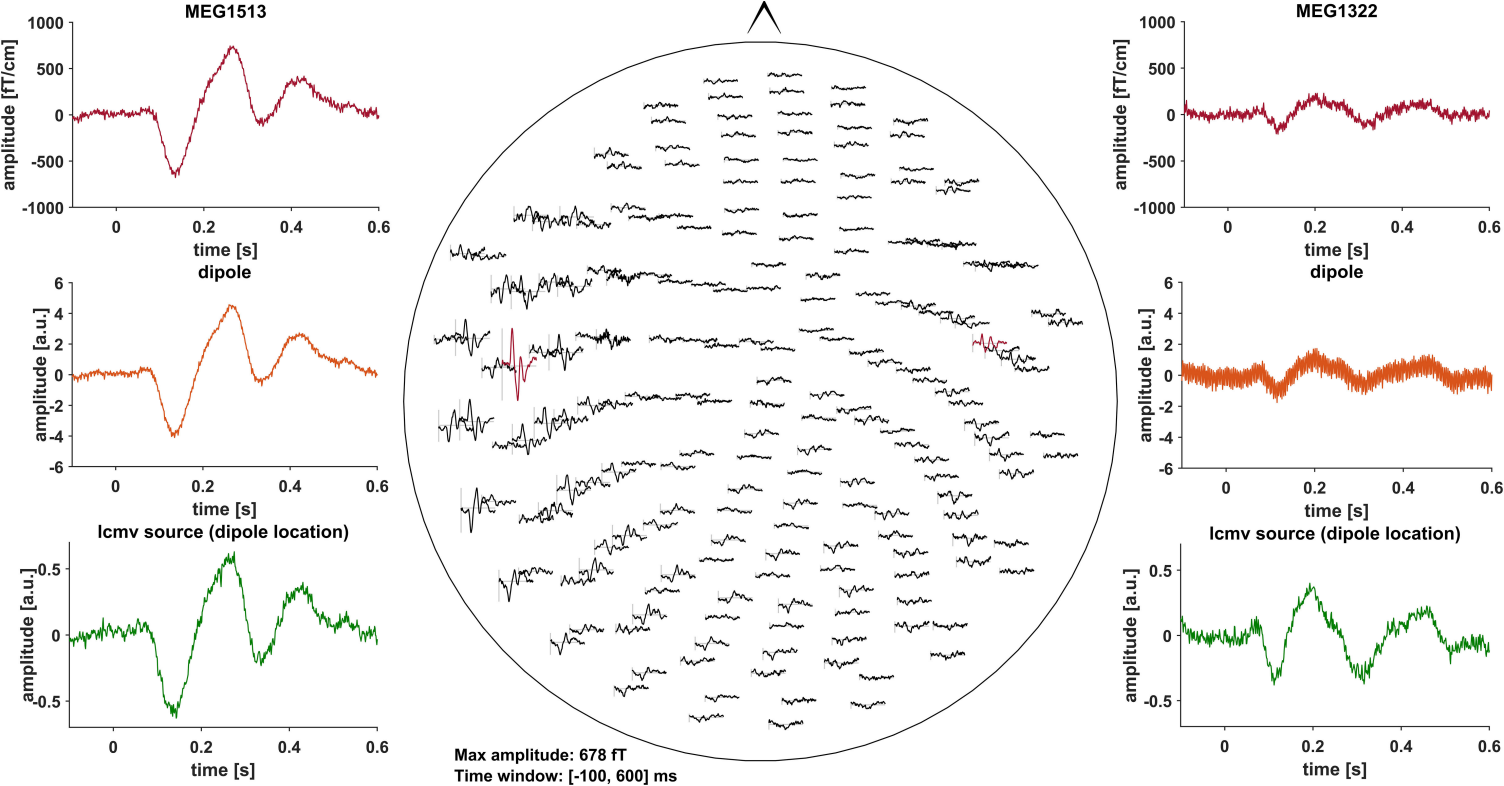
All of the graphs show averaged ERF for patient # 13 (stimulation ON). Middle: whole head 40 Hz low-pass filtered MEG channel overview without DBS artifact contamination. Channels selected to fit a dipole are marked red and shown in the top left- and right-hand corners (MEG1513, MEG1322). Comparison of sensor level (red) and two different source localization approaches: dipole (orange) and LCMV beamforming (green). After fitting the dipole, the source time series of its corresponding vertex in the LCMV beamformer source model was extracted (green). Dipole fitting was not part of the analysis and only conducted in this patient for visualization purposes.

**Figure 2:**
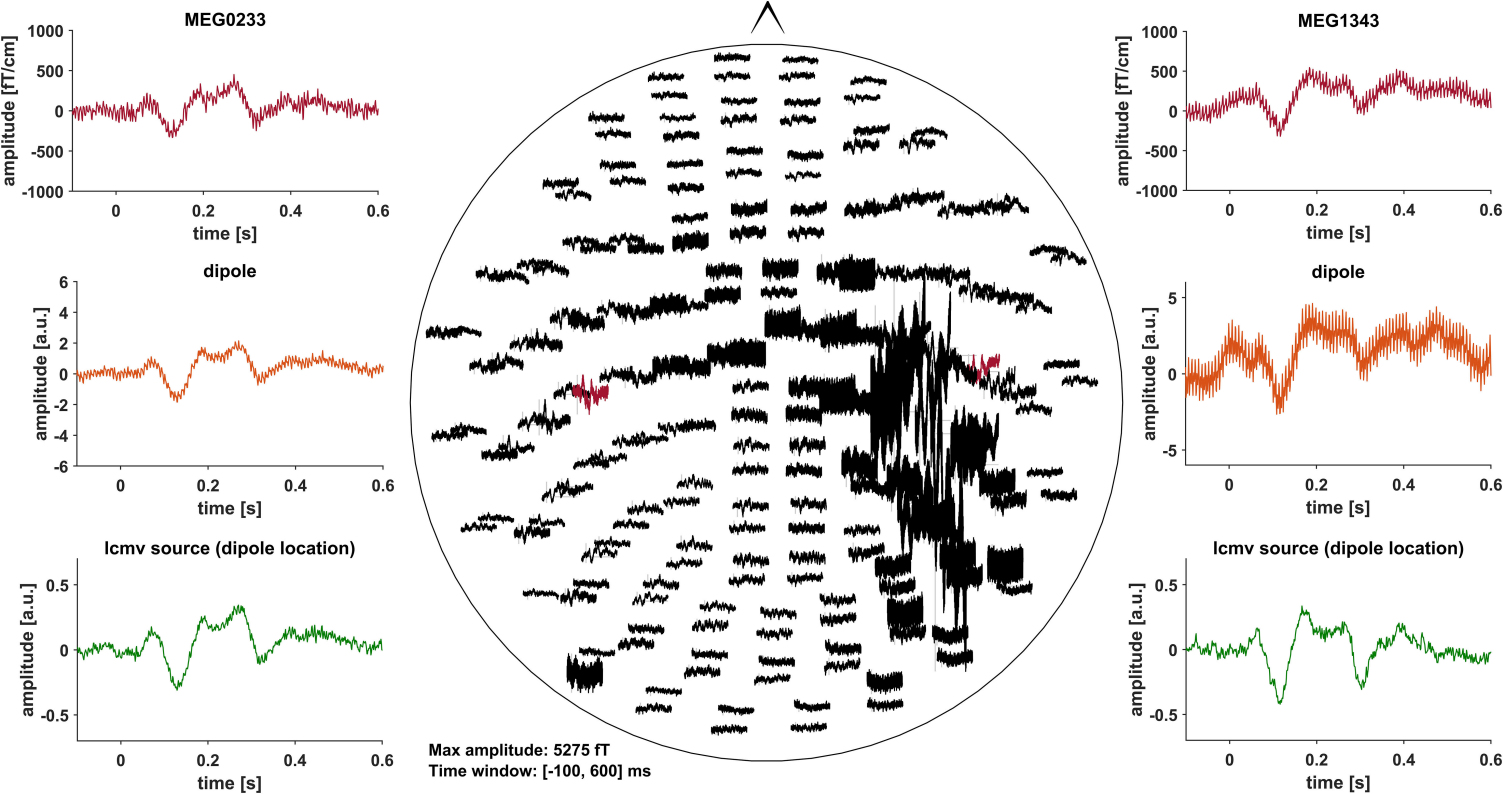
All of the graphs show unfiltered averaged ERF for patient # 16 (stimulation ON). Middle: whole head unfiltered MEG channel overview with strong focal artifact contamination in channels over the right skull. Channels that were selected to fit a dipole are marked red and shown in the top left- and right-hand corners (MEG0233, MEG1343). Comparison of sensor level (red) and two different source localization approaches: dipole (orange) and LCMV beamforming (green). After fitting the dipole, the source time series of its corresponding vertex in the LCMV beamformer source model was extracted (green). Dipole fitting was not part of the analysis and only conducted in this patient for visualization purposes.

Event related fields (ERF) were previously demonstrated to be nearly unaffected by DBS induced artifacts, due to the fact that DBS pulses are not time-locked to the stimulus (30). Artifacts induced by the DBS hardware, however, might affect ERF (31). To minimize these artifacts, we pre-selected PD patients with DBS systems using only slightly or non-magnetic hardware components at the skull, i.e., DBS systems by Abbott® with their low-iron extension cable or DBS systems by Boston Scientific® (Table 1). Indeed, in most cases, no signs of artifact contamination were visible after averaging and 40 Hz low-pass filtering at the channel level (Figure 1). Still, in about 5 patients there were focal low and high frequency artifacts in channels over the right side of the skull, even after averaging (Figure 2). Therefore, we worked with Linearly Constrained Minimum Variance (LCMV) beamforming, which was demonstrated to reduce artifacts caused by movements of the magnetic DBS hardware components (32). Even unfiltered, the obtained ERF from the source level presented no signs of artifact contamination after LCMV beamforming (Figure 2).

Signal space projection (SSP) as implemented in Brainstorm was used to eliminate cardiac artifacts. Then, we inspected and removed trials affected by eye movement, muscle, and sensor artifacts. Trials resulting in negative response magnitudes in the vocal response analysis were excluded (Supplementary Table 1). In total, an average of 25.69% of trials were omitted for OFF and 25.19% of trials for ON. The mean number of averaged trials was 93.3 for OFF stimulation (95%-CI [71.4, 115.2]) and 94.6 for ON stimulation (95%-CI [76.14, 113.1]) also for vocal response analysis (Supplementary Table 1). Clean trials were averaged and projected to the individual anatomical source level using LCMV beamformer (33). For source reconstruction, individual anatomical cortical surfaces were used and an overlapping sphere head model was constructed in Brainstorm. The anatomical surfaces were extracted from clinical MRIs, which every patient had received before DBS surgery, using Freesurfer (http://surfer.nmr.mgh.harvard.edu/). A z-score baseline normalization (−100 to −1 ms) was applied to the individual source level data. Baseline noise of averaged trials before source localization is given in Supplementary Table 2. Next, all individual source level data were projected to MNI space using Freesurfer's registered spheres (34).

The analysis focused on four regions of interest in the right and left hemisphere, previously described to relate to P200 changes in PD (13): (i) auditory cortex (left AC, [−54.3, −26.5, 11.6]; right AC, [54.3, −26.5, 11.6]), (ii) superior temporal gyrus (left STG, [−58, −19.8, −6]; right STG, [58, −19.8, −6]), (iii) inferior parietal lobe (left IPL, [−51.2, −43.3, 40.0]; right IPL, [51.2, −43.3, 40.0]), and (iv) premotor cortex (left PMC, [−46, 0, 35]; right PMC, [46, 0, 35]). To this end, we used predefined MNI coordinates, representing the respective center of mass and enlarged the region by 15 vertices around the coordinate point (19). To extract individual time series for each region, a principal component analysis (PCA) was conducted. The first principal component was selected, resulting in one time series for each patient and condition. Data was then low-pass filtered (40 Hz) and time series with positive N100m peaks were flipped. Thus, in every acoustically evoked field (AEF), the N100m was a negative peak. Sign flipping was necessary to deal with sign ambiguity of MEG data. Afterwards, we automatically detected the minima of N100m (100–200 ms) and P200m maxima (200–300 ms) and used these for statistical analysis.

### Statistics

SPSS (v.25.0) was used for the statistical analyses of both behavioral and neurophysiological data. To test for differences between ON and OFF stimulation, the magnitudes of vocal responses as well as voice jitter and voice intensity were subjected to paired *t*-tests. To explore the relation between vocal response magnitude and pitch variability, we calculated Pearson's *r*, separately for ON and OFF stimulation. Repeated-measures Analyses of Variance (RM-ANOVA) were conducted to analyze differences between ERF amplitudes and latencies (N100m and P200m). Here, task (vocal vs. listen) and stimulation condition (ON vs. OFF) were within-subject factors. Finally, we calculated Cohen's *d*_z_ for each *t*-test and the effect size *f* for each of the RM-ANOVAs using release 3.1.9.4 of G^*^Power (35).

## Results

### Behavioral Data

**Figure 3:**
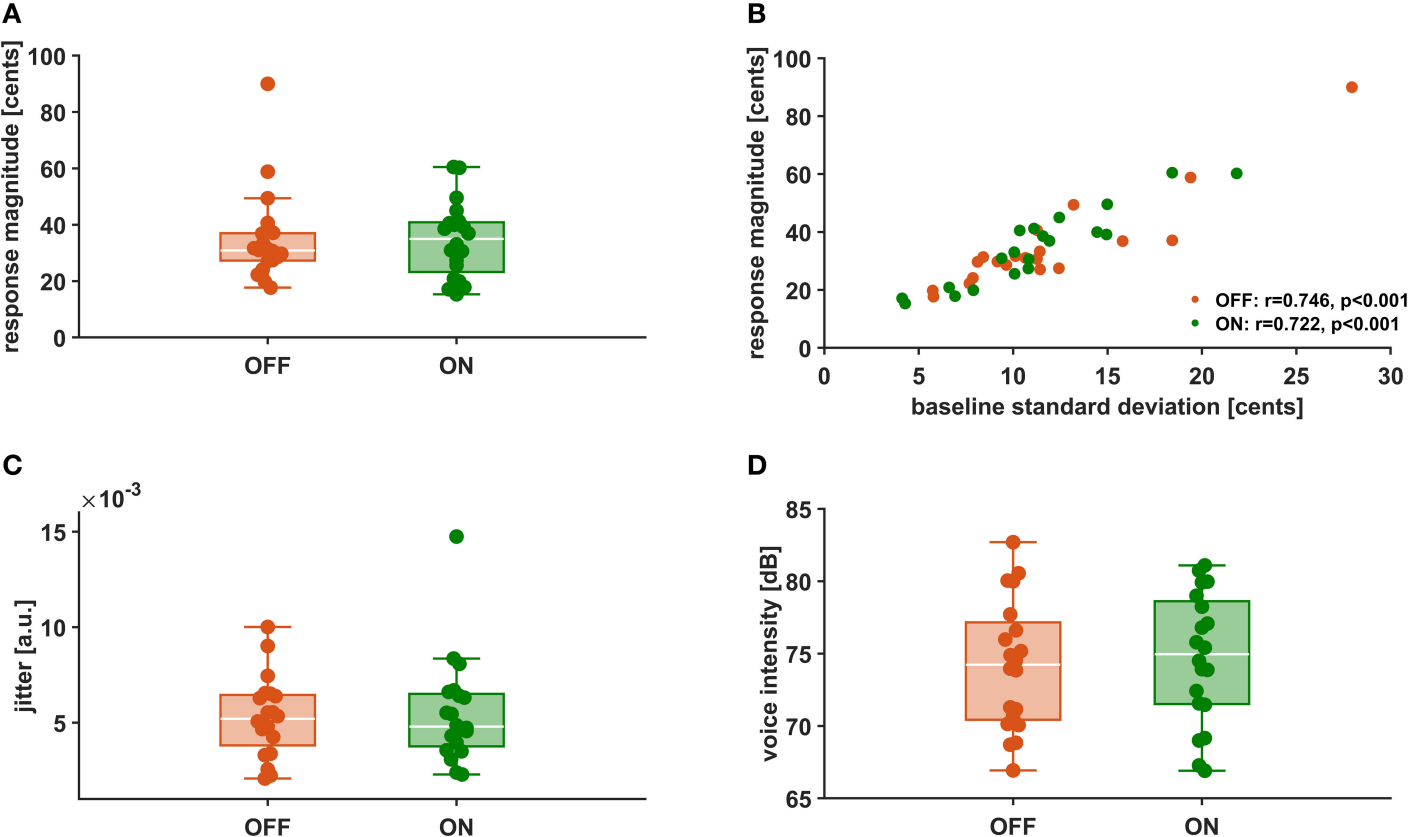
Vocal f0 response magnitude **(A)**, Correlation between vocal f0 response magnitude and f0 baseline standard deviation **(B)**, voice intensity **(C)**, and voice jitter **(D)** during stimulation OFF (orange) and ON (green).

In Figures 3A,C,D the distributions of mean vocal f0 response magnitudes to pitch-shifted feedback, voice intensity as well as voice jitter are depicted for stimulation ON and stimulation OFF. There was no statistically significant difference between stimulation conditions for vocal response magnitudes [OFF: 23.91 ± 10.44 cents, ON: 24.41 ± 8.11 cents, *t*_(19)_ = −0.245, *p* = 0.809, *d* = −0.055]. Also for voice intensity [OFF: 74.7 ± 4.5 dB, ON: 74.2 ± 4.5 dB, *t*_(19)_ = −0.826, *p* = 0.419, *d* = −0.185] and jitter [OFF: 0.0053 ± 0.0021, ON:0.0055 ± 0.0028, *t*_(19)_ = −0.437, *p* = 0.667, *d* = −0.098] no differences could be observed between stimulation conditions. Though, in both conditions, there was a positive correlation (OFF: *r* = 0.746, *p* < 0.001, ON: *r* = 0.722, *p* < 0.001) between vocal response magnitude and pitch variability (Figure 3B).

### Neurophysiological Data

**Figure 4:**
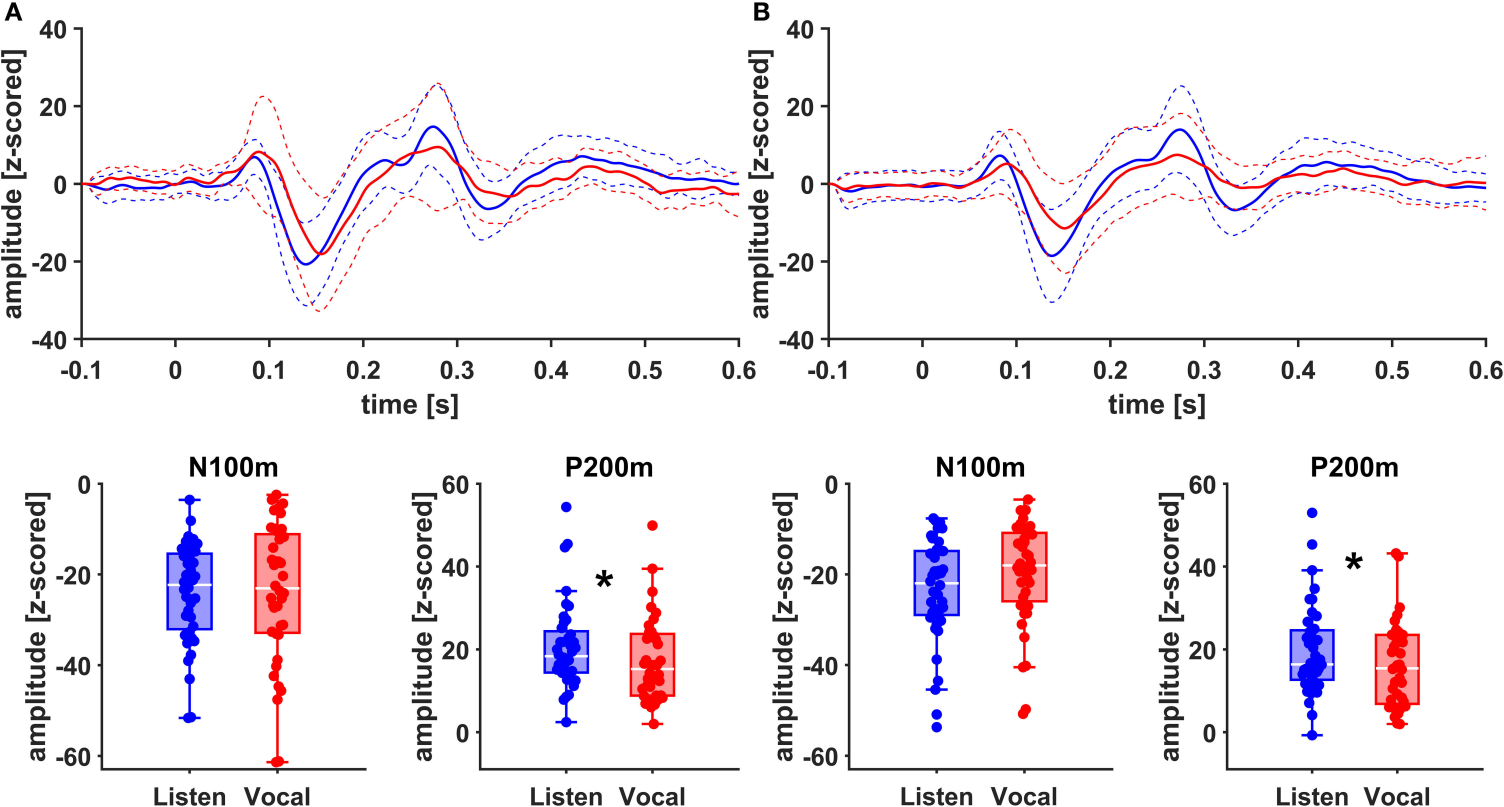
Grand Averages (*n* = 20) of event related fields extracted as principal components from left AC **(A)** and left STG **(B)** time-locked to pitch-shifting onset, listen (blue) vs. vocalization (red). Dashed lines represent standard deviations. Below: Boxplots of N100m and P200m amplitudes for each left AC and STG, listen (blue) vs. vocalization (red). DBS ON and DBS OFF data was pooled for these figures. Asterisks mark significant (α < 0.05) main effects of task between P200m amplitudes.

**Figure 5:**
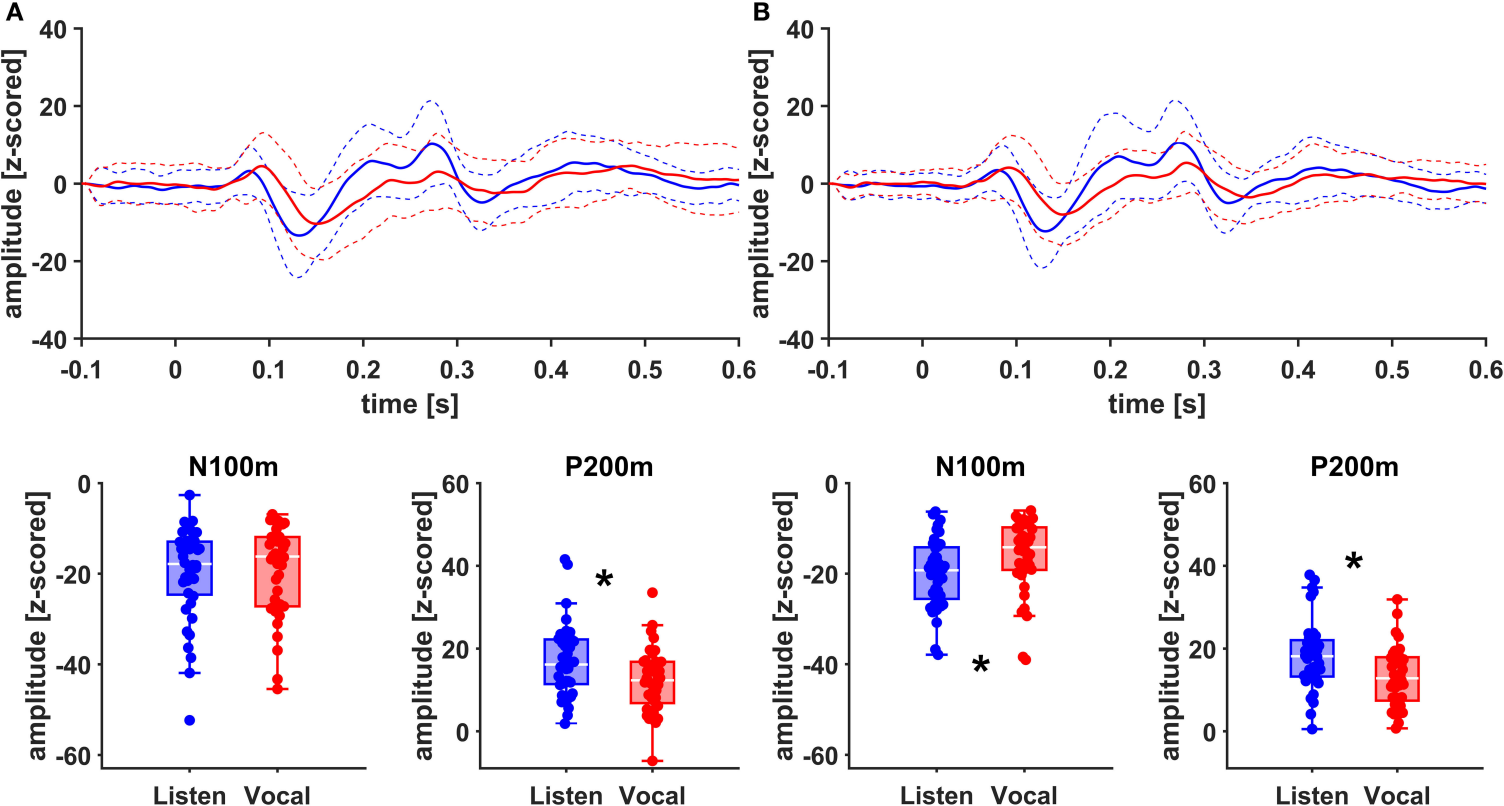
Grand Averages (*n* = 20) of event related fields extracted as principal components from right AC **(A)** and right STG **(B)** time-locked to pitch-shifting onset, listen (blue) vs. vocalization (red). Dashed lines represent standard deviations. Below: Boxplots of N100m and P200m amplitudes for each right AC and STG, listen (blue) vs. vocalization (red). DBS ON and DBS OFF data was pooled for these figures. Asterisks mark significant (α < 0.05) main effects of task between N100m and P200m amplitudes.

There was no significant main effect of stimulation on cortical ERF amplitudes [left AC N100m: *F*_(1, 19)_ = 0.337, *p* = 0.586, *f* = 0.133; left AC P200m: *F*_(1, 19)_ = 0.433, *p* = 0.518, *f* = 0.151]. However, when comparing P200m amplitudes between tasks (vocal vs. listen), significant differences were observed [left AC P200m: *F*_(1, 19)_ = 10.241, *p* = 0.005, *f* = 0.734; right STG P200m: *F*_(1, 19)_ = 8.393, *p* = 0.009, *f* = 0.664; Figures 4, 5]. *Post-hoc* paired *t*-tests revealed a significant difference between tasks for P200m amplitudes over right and left STG and AC, when the stimulation was ON [left AC: *t*_(19)_ = 2.66, *p* = 0.015, *d* = 0.597; right STG *t*_(19)_ = 6.47, *p* = 0.006, *d* = 0.689]. Thus, the left and right AC and STG P200m amplitudes were larger in the listen task compared to the vocal task. Additionally, the right STG showed a main effect of task (vocal vs. listen) for the N100m amplitudes as well [N100m: *F*_(1, 19)_ = 8.026, *p* = 0.011, *f* = 0.650]. *Post-hoc* testing revealed a difference between N100m amplitudes for stimulation ON [*t*_(19)_ = −5.25, *p* = 0.009, *d* = −0.649]. RM-ANOVA evaluating latency differences between task and stimulation conditions showed longer latencies for vocalization than listening over left and right STG and AC as well as left PMC [right AC: N100m: *F*_(1, 19)_ = 9.069, *p* = 0.007, *f* = 0.691; right STG: N100m: *F*_(1, 19)_ = 11.210, *p* = 0.003, *f* = 0.768]. *Post-hoc* analysis revealed longer latencies of the right AC ERF during vocalization, both for stimulation OFF and ON [OFF listen vs. vocal: *t*_(19)_ = −0.013, *p* = 0.044, *d* = −0.483; ON listen vs. vocal: *t*_(19)_ = −0.017, *p* = 0.019, *d* = −0.573]. The RM-ANOVA main effect results for all ROI and the *post-hoc* paired *t*-test results are summarized in Tables 2, 3. ERF amplitudes and latencies for all conditions are summarized in Supplementary Tables 3, 4.

**Table 2:** RM-ANOVA results over all ROI of both hemispheres comparing N100m and P200m ERF amplitudes between the within-subject factors task (vocal vs. listen) and stimulation (ON vs. OFF).

**N100m and P200m amplitudes (RM-ANOVA results)**					
**Left hemisphere**			**Right hemisphere**		
**Region**	**Task (vocal vs. listen)**	**Stimulation (ON vs. OFF)**	**Region**	**Task (vocal vs. listen)**	**Stimulation (ON vs. OFF)**
AC	N100m: *F*_(1, 19)_ = 0.188, *p* = 0.670, *f* = 0.099	N100m: *F*_(1, 19)_ = 0.337, *p* = 0.568, *f* = 0.133	AC	N100m: *F*_(1, 19)_ = 0.032, *p* = 0.860, *f* = 0.045	N100m: *F*_(1, 19)_ = 0.298, *p* = 0.591, *f* = 0.123
	P200m: *F*_(1, 19)_ = 10.241, ***p*** **= 0.005**, *f* = 0.734	P200m: *F*_(1, 19)_ = 0.433, *p* = 0.518, *f* = 0.151		P200m: *F*_(1, 19)_ = 6.849, ***p*** **= 0.017**, *f* = 0.600	P200m: *F*_(1, 19)_ = 0.319, *p* = 0.579, *f* = 0.132
STG	N100m: *F*_(1, 19)_ = 3.368, *p* = 0.082, *f* = 0.421	N100m: *F*_(1, 19)_ = 0.002, *p* = 0.969, *f* = 0.009	STG	N100m: *F*_(1, 19)_ = 8.026, ***p*** **= 0.011**, *f* = 0.650	N100m: *F*_(1, 19)_ = 0.614, *p* = 0.443, *f* = 0.179
	P200m: *F*_(1, 19)_ = 5.758, ***p*** **= 0.027**, *f* = 0.551	P200m: *F*_(1, 19)_ = 0.123, *p* = 0.729, *f* = 0.081		P200m: *F*_(1, 19)_ = 8.393, ***p*** **= 0.009**, *f* = 0.664	P200m: *F*_(1, 19)_ = 0.187, *p* = 0.670, *f* = 0.100
PMC	N100m: *F*_(1, 19)_ = 1.199, *p* = 0.287, *f* = 0.251	N100m: *F*_(1, 19)_ = 1.095, *p* = 0.309, *f* = 0.240	PMC	N100m: *F*_(1, 19)_ = 0.343, *p* = 0.565, *f* = 0.135	N100m: *F*_(1, 19)_ = 1.301, *p* = 0.268, *f* = 0.261
	P200m: *F*_(1, 19)_ = 0.053, *p* = 0.821, *f* = 0.053	P200m: *F*_(1, 19)_ = 0.481, *p* = 0.497, *f* = 0.159		P200m: *F*_(1, 19)_ = 5.825, ***p*** **= 0.026**, *f* = 0.554	P200m: *F*_(1, 19)_ = 0.017, *p* = 0.897, *f* = 0.032
IPL	N100m: *F*_(1, 19)_ = 0.038, *p* = 0.848, *f* = 0.045	N100m: *F*_(1, 19)_ = 0.025, *p* = 0.875, *f* = 0.036	IPL	N100m: *F*_(1, 19)_ = 0.086, *p* = 0.772, *f* = 0.071	N100m: *F*_(1, 19)_ = 1.724, *p* = 0.205, *f* = 0.301
	P200m: *F*_(1, 19)_ = 4.399, *p* = 0.050, *f* = 0.481	P200m: *F*_(1, 19)_ = 1.204, *p* = 0.286, *f* = 0.252		P200m: *F*_(1, 19)_ = 2.475, *p* = 0.132, *f* = 0.360	P200m: *F*_(1, 19)_ = 0.023, *p* = 0.882, *f* = 0.032
***Post-hoc*** **tests**			***Post-hoc*** **tests**		
AC P200m	OFF listen – OFF vocal = 2.66, 95%-CI [−5.67, 10.98], *p* = 0.512, *d* = 0.149		AC P200m	OFF listen – OFF vocal = 4.12, 95%-CI [−1.15, 9.40], *p* = 0.118, *d* = 0.366	
	ON listen – ON vocal = 13.28, 95%-CI [2.98, 24.65], ***p*** **= 0.015**, *d* = 0.597			ON listen – ON vocal = 5.63, 95%-CI [1.59, 9.67], ***p*** **= 0.009**, *d* = 0.652	
STG P200m	OFF listen – OFF vocal = 2.31, 95%-CI [−2.04, 6.66], *p* = 0.280, *d* = 0.248		STG N100m	OFF listen – OFF vocal = −2.59, 95%-CI [−7.20, 2.01], *p* = 0.253, *d* = −0.263	
	ON listen – ON vocal = 6.14, 95%-CI [0.53, 11.75], ***p*** **= 0.034**, *d* = 0.512			ON listen – ON vocal = −5.25, 95%-CI [−9.03, −1.46], ***p*** **= 0.009**, *d* = −0.649	
			STG P200m	OFF listen – OFF vocal = 4.06, 95%-CI [−1.64, 9.77], *p* = 0.153, *d* = 0.333	
				ON listen – ON vocal = 6.47, 95%-CI [2.08, 10.87], ***p*** **= 0.006**, *d* = 0.689	
			PMC P200m	OFF listen – OFF vocal = 2.96, 95%-CI [−0.04, 5.96], *p* = 0.053, *d* = 0.462	
				ON listen – ON vocal = 1.61, 95%-CI [−0.86, 4.08], *p* = 0.190, *d* = 0.305	

*Post-hoc paired t-test results are displayed in the lower parts of the table. Significant p-values (α < 0.05) in bold*.

**Table 3:** RM-ANOVA results over all ROI of both hemispheres comparing N100m and P200m ERF latencies between the within-subject factors task (vocal vs. listen) and stimulation (ON vs. OFF).

**N100m and P200m latencies (RM-ANOVA results)**					
**Left hemisphere**			**Right hemisphere**		
**Region**	**Task (vocal vs. listen)**	**Stimulation (ON vs. OFF)**	**Region**	**Task (vocal vs. listen)**	**Stimulation (ON vs. OFF)**
AC	N100m: *F*_(1, 19)_ = 5.071, ***p*** **= 0.036**, *f* = 0.517	N100m: *F*_(1, 19)_ = 3.299, *p* = 0.085, *f* = 0.417	AC	N100m: *F*_(1, 19)_ = 9.069, ***p*** **= 0.007**, *f* = 0.691	N100m: *F*_(1, 19)_ = 0.192, *p* = 0.666, *f* = 0.101
	P200m: *F*_(1, 19)_ = 0.363, *p* = 0.554, *f* = 0.138	P200m: *F*_(1, 19)_ = 0.003, *p* = 0.96, *f* = 0.012		P200m: *F*_(1, 19)_ = 3.044, *p* = 0.097, *f* = 0.400	P200m: *F*_(1, 19)_ = 0.359, *p* = 0.556, *f* = 0.139
STG	N100m: *F*_(1, 19)_ = 5.791, ***p*** **= 0.026**, *f* = 0.553	N100m: *F*_(1, 19)_ = 0.356, *p* = 0.558, *f* = 0.135	STG	N100m: *F*_(1, 19)_ = 11.210, ***p*** **= 0.003**, *f* = 0.768	N100m: *F*_(1, 19)_ = 5.644, ***p*** **= 0.028**, *f* = 0.545
	P200m: *F*_(1, 19)_ = 0.407, *p* = 0.531, *f* = 0.146	P200m: *F*_(1, 19)_ = 0.681, *p* = 0.419, *f* = 0.19		P200m: *F*_(1, 19)_ = 4.327 *p* = 0.051, *f* = 0.476	P200m: *F*_(1, 19)_ = 7.852, ***p*** **= 0.011**, *f* = 0.642
PMC	N100m: *F*_(1, 19)_ = 4.984, ***p*** **= 0.038**, *f* = 0.512	N100m: *F*_(1, 19)_ = 0.312, *p* = 0.583, *f* = 0.128	PMC	N100m: *F*_(1, 19)_ = 3.161, *p* = 0.091, *f* = 0.408	N100m: *F*_(1, 19)_ = 0.002, *p* = 0.967, *f* = 0.010
	P200m: *F*_(1, 19)_ = 0.001, *p* = 0.971, *f* = 0.008	P200m: *F*_(1, 19)_ = 0.324, *p* = 0.576, *f* = 0.132		P200m: *F*_(1, 19)_ = 2.050, *p* = 0.168, *f* = 0.328	P200m: *F*_(1, 19)_ = 0.972, *p* = 0.337, *f* = 0.227
IPL	N100m: *F*_(1, 19)_ = 0.009, *p* = 0.925, *f* = 0.022	N100m: *F*_(1, 19)_ = 0.221, *p* = 0.644, *f* = 0.108	IPL	N100m: *F*_(1, 19)_ = 4.630, ***p*** **= 0.044**, *f* = 0.494	N100m: *F*_(1, 19)_ = 0.529, *p* = 0.476, *f* = 0.167
	P200m: *F*_(1, 19)_ = 1.139, *p* = 0.299, *f* = 0.245	P200m: *F*_(1, 19)_ = 0.008, *p* = 0.928, *f* = 0.021		P200m: *F*_(1, 19)_ = 4.208, *p* = 0.054, *f* = 0.470	P200m: *F*_(1, 19)_ = 1.139, *p* = 0.299, *f* = 0.246
***Post-hoc*** **tests**			***Post-hoc*** **tests**		
AC N100m	OFF listen – OFF vocal = −0.021, 95%-CI [−0.036, −0.007], ***p*** **= 0.007**, *d* = −0.682		AC N100m	OFF listen – OFF vocal = −0.013, 95%-CI [−0.025, −0.0004], ***p*** **= 0.044**, *d* = −0.483	
	ON listen – ON vocal = −0.008, 95%-CI [−0.028, −0.012], *p* = 0.421, *d* = −0.184			ON listen – ON vocal = −0.017, 95%-CI [−0.03, −0.003], ***p*** **= 0.019**, *d* = −0.573	
STG N100m	OFF listen – OFF vocal = −0.01, 95%-CI [−0.025, 0.004], *p* = 0.142, *d* = −0.343		STG N100m	OFF listen – OFF vocal = −0.013, 95%-CI [−0.026, 0.001], *p* = 0.068, *d* = −0.432	
				ON listen – ON vocal = −0.026, 95%-CI [−0.046, −0.007], ***p*** **= 0.010**, *d* = −0.635	
	ON listen – ON vocal = −0.013, 95%-CI [−0.026, 0], *p* = 0.058, *d* = −0.452			OFF listen – ON listen = −0.001, 95%-CI [−0.014, 0.012], *p* = 0.856, *d* = −0.041	
				OFF vocal – ON vocal = −0.015, 95%-CI [−0.029, −0.001], ***p*** **= 0.037**, *d* = −0.500	
PMC N100m	OFF listen – OFF vocal = −0.008, 95%-CI [−0.027, 0.01], *p* = 0.364, *d* = −0.208		STG P200m	OFF listen – ON listen = −0.009, 95%-CI [−0.03, 0.012], *p* = 0.397, *d* = −0.194	
	ON listen – ON vocal = −0.018, 95%-CI [−0.033, −0.003], ***p*** **= 0.024**, *d* = −0.548			OFF vocal – ON vocal = −0.023, 95%-CI [−0.041, −0.005], ***p*** **= 0.013**, *d* = −0.614	
			IPL N100m	OFF listen – OFF vocal = −0.013, 95%-CI [−0.028, 0.002], *p* = 0.095, *d* = −0.393	
				ON listen – ON vocal = −0.013, 95%-CI [−0.03, 0.004], *p* = 0.122, *d* = −0.362	

*Post-hoc paired t-test results are displayed in the lower parts of the table. Significant p-values (α < 0.05) in bold*.

## Discussion

In this study, we investigated the effect of DBS on auditory-motor integration of speech. While we could not find an effect of subthalamic DBS on vocal compensation to pitch-shifted feedback, there was a positive correlation between vocal response magnitudes and pitch variability in both conditions. In line with the behavioral findings, a difference between ERF amplitudes, comparing ON and OFF stimulation, was not observed. However, when looking at differences between vocalization and listening, amplitudes were larger and latencies shorter for listening over right and left AC and STG.

### Auditory-Motor Integration Is Not Modulated by DBS

Analyzing voice recordings in the stimulation ON and OFF, we found vocal response magnitudes opposing the downward pitch-shifted feedback of about +24 cents, which is similar to results of earlier studies with this experimental design (12, 13). In addition, we could replicate the positive correlation between vocal response magnitude and pitch variability (12, 13). This means, the stronger a patient compensated to pitch-shifted feedback, the larger was their own vocal pitch variability. This observation tallies with earlier work and is probably related to deficits in the mechanisms of auditory-motor integration, as it was only observed in patients (12, 13). Noteworthy, the positive correlation of these two parameters—f0 response and f0 variability—was similar with DBS ON and OFF (Figure 3B). This suggests that the deficits underlying this relation were not modulated by subthalamic DBS. The fact that we could not find a difference between vocal responses in the stimulation ON vs. OFF supports this notion further.

In a recent study, subthalamic DBS was shown to attenuate compensating vocal response magnitudes to pitch-shifted feedback and also improved voice jitter (4). However, these results were solely based on 10 PD patients. Earlier work already suggested that DBS effects on acoustic parameters are highly individual (3). Thus, Skodda et al. could only find tendencies of amelioration of pitch variability and concluded that DBS effects on Parkinsonian speech differ considerably between patients. With the present findings based on 20 PD patients, we neither observed an effect on vocal nor neurophysiological responses. Additionally, we could not identify any clinical or acoustical parameter predicting individual performances. Thus, DBS might have critical limitations when it comes to influencing the modulation of speech in PD. One parameter, which we did not include in our analysis, however, is electrode placement. A recent study demonstrated that electrode placement in the anterior portion of the STN was associated with an improvement of voice-related outcomes in PD patients (36). Future studies investigating larger patient samples should assess, whether differences in individual speech performance and modulation of speech can be explained by the electrode location.

### Vocalization Induced Suppression

In accordance with our behavioral findings, we could not see a significant difference between ERF amplitudes, comparing ON and OFF stimulation. Still, the ERF amplitudes were larger in the listen task than in the vocal task over the right and left AC and STG (Figures 4, 5). These results seem to contradict earlier findings, where a so-called vocalization-induced enhancement of P200 amplitudes was reported for healthy individuals and was even augmented in PD (13, 18). Within a previous EEG experiment, the P200 response for the vocalization task was increased over the Cz electrode (13). The P200 peak for the vocalization task was followed by a sustained amplitude plateau. This plateau might be interpreted as a P300 component combined with an enhanced P200 response. However, MEG normally fails to represent magnetic field P300 equivalents due to the deep localization of their generators (37). Indeed, a MEG study examining vocalization-induced enhancement in 11 healthy individuals could not find an enlargement of P200m amplitudes as clear as in the EEG experiment (19). To solve the issue of limited comparability between MEG and EEG findings, experiments focusing on late auditory potentials should probably rather be conducted with high-density EEG measurements or a combination of EEG and MEG.

Since we assessed responses to pitch changes in self-generated speech and P200 changes in PD relate to a left-lateralized network (13), we expected changes to be localized mainly to the left hemisphere. Indeed, amplitudes appeared to be higher in the left hemisphere (Figure 4). However, when comparing effect sizes of left and right STG, there is a stronger main effect of task (vocal vs. listen) for the right STG [right STG: P200m: *F*_(1, 19)_ = 8.393, *p* = 0.009, *f* = 0.664; left STG: P200m: *F*_(1, 19)_ = 5.758, *p* = 0.027, *f* = 0.551]. Additionally, there is robust evidence concerning vocalization-induced suppression, especially for N100m amplitudes, probably reflecting auditory cortex sensitivity to self-generated sounds (18, 19, 38). The right AC is known to be especially sensitive to the spectral dimension of sound (39). In line with these observations, N100m amplitudes were suppressed during vocalization at the right STG (Figure 4). Similarly, N100m latencies were longer during vocalization at left and right AC and STG as well as left PMC, which has been described before (13, 38).

### DBS Artifacts

Measuring brain activity during active DBS using MEG is an emerging field of research (40). As DBS-MEG recordings are associated with more or less severe artifacts, the use of artifact reduction methods is most often necessary (30, 41). In case it is not necessary, however, these methods should not be applied because they bear the risk of altering brain signals, e.g., amplitude reduction (30). Here, we investigated ERF, which are comparably robust to DBS artifacts (Figure 1). Moreover, using LCMV beamforming, we reduced artifacts caused by the movement of ferromagnetic DBS components additionally (32) (Figure 2). Due to the fact that the source level DBS ON data revealed similar ERFs as DBS OFF data, we can assume that the stimulation artifact itself was sufficiently reduced with that approach. These findings might therefore facilitate and pave the way for further investigations on ERFs during DBS to better understand the cortical effects of DBS. The use of recent more noise-resistant SQUIDs in newer MEG systems might even further improve data quality in future combined MEG-DBS-studies.

## Conclusion

Auditory-motor deficits play an important role for Parkinsonian speech pathology and are represented by strong pitch compensations to pitch-shifted auditory feedback correlating with pitch variability. Subthalamic DBS appears not to modulate these compensations in PD and therefore seems to have no substantial effect on the auditory-motor integration of speech. Moreover, we were able to demonstrate that it is possible to explore auditory ERFs in DBS patients using LCMV beamforming without additional artifact reduction methods.

## Data Availability Statement

The raw data supporting the conclusions of this article will be made available by the authors, without undue reservation.

## Ethics Statement

The studies involving human participants were reviewed and approved by Medical Faculty's Ethics Committee Heinrich-Heine University Düsseldorf Germany. The patients/participants provided their written informed consent to participate in this study.

## Author Contributions

BB, JR, HK, AS, and MB contributed conception and design of the study. BB, EF, HK, JR, MB, and JH contributed to and developed the methodology and conducted parts of the formal and statistical analysis. RV developed the voice analysis scripts and wrote parts of the methods section. BB, AS, and MB recruited patients and conducted the measurements. BB and HK organized the database. BB wrote the first draft of the manuscript. MB, EF, JH, RV, and JR wrote sections of the manuscript. All authors contributed to manuscript revision, read, and approved the submitted version.

## Conflict of Interest

AS has received consultant/speaker fees from Boston Scientific, Medtronic, and Abbott. The remaining authors declare that the research was conducted in the absence of any commercial or financial relationships that could be construed as a potential conflict of interest.
