# Supplementary material for: Deep Brain Stimulation Does Not Modulate Auditory-Motor Integration of Speech in Parkinson's Disease
Source: Front Neurol. 2020 Jul 10;11:655. doi: 10.3389/fneur.2020.00655 (PMC7366847; doi:10.3389/fneur.2020.00655)
Supplement: Supplementary file 1 [file Data_Sheet_1.PDF]

# *Supplementary Material*

## 1 Supplementary Tables

**Supplementary Table 1.** Number of trials per patient ON and OFF stimulation. Trials = total number of trials before artefact cleaning. Clean trials = number of trials after MEG artefact cleaning. Up trials= number of trials after MEG artefact cleaning, only taking into account trials with an upward vocal compensation to downward pitch shifting. The number of up trials equals the number of trials averaged per patient.

| Patient | trials OFF | trials ON | clean trials OFF | clean trials ON | up trials OFF | up trials ON |
|---|---|---|---|---|---|---|
| **1** | 101 | 112 | 84 | 87 | 80 | 80 |
| **2** | 141 | 138 | 118 | 119 | 114 | 118 |
| **3** | 121 | 121 | 90 | 96 | 90 | 94 |
| **4** | 137 | 139 | 107 | 112 | 99 | 103 |
| **5** | 122 | 119 | 101 | 100 | 101 | 98 |
| **6** | 77 | 89 | 44 | 59 | 44 | 54 |
| **7** | 121 | 115 | 90 | 88 | 88 | 86 |
| **8** | 140 | 134 | 99 | 98 | 99 | 94 |
| **9** | 107 | 98 | 68 | 64 | 67 | 64 |
| **10** | 109 | 122 | 89 | 102 | 80 | 101 |
| **11** | 87 | 111 | 69 | 84 | 61 | 73 |
| **12** | 93 | 112 | 76 | 79 | 68 | 75 |
| **13** | 145 | 152 | 102 | 122 | 102 | 116 |
| **14** | 128 | 113 | 103 | 94 | 102 | 93 |
| **15** | 161 | 148 | 132 | 118 | 132 | 101 |
| **16** | 141 | 142 | 116 | 120 | 114 | 120 |
| **17** | 157 | 162 | 132 | 117 | 120 | 111 |
| **18** | 132 | 118 | 112 | 98 | 88 | 87 |
| **19** | 147 | 150 | 115 | 116 | 114 | 113 |
| **20** | 144 | 134 | 117 | 115 | 103 | 111 |
| **Mean** | **125.55** | **126.45** | **98.20** | **99.40** | **93.30** | **94.60** |
| **SD** | **23.47** | **19.11** | **22.43** | **18.53** | **21.88** | **18.46** |

**Supplementary Table 2.** Baseline noise (fT/cm) for all conditions and all subjects over all channels before baseline normalization was applied. Mean baseline noise (fT/cm) and standard deviations are given at the bottom of the table.

| Patient | OFF listen | OFF vocal | ON listen | ON vocal |
|---|---|---|---|---|
| **1** | -28,48 | -85,75 | -82,67 | 149,01 |
| **2** | -80,58 | -1988,47 | -3820,79 | -3197,89 |
| **3** | 20,58 | -299,09 | -29,38 | 83,78 |
| **4** | 7,28 | 62,33 | 22,89 | 186,41 |
| **5** | 10,64 | 95,2 | -191,52 | 154,65 |
| **6** | 233,82 | 233,82 | -33,58 | 126,43 |
| **7** | -4,51 | 21,14 | -0,07 | 88,36 |
| **8** | -37,77 | -23,11 | -2351,2 | -2255,03 |
| **9** | 101,25 | -4804,16 | 303,86 | -994,23 |
| **10** | 121,38 | 68,96 | 2,43 | 26,66 |
| **11** | -28,08 | 137,02 | 43,46 | -206,8 |
| **12** | -0,01 | -42,56 | 0,26 | 11,03 |
| **13** | 23,84 | 653,25 | -106,51 | 1440,68 |
| **14** | -70,71 | 595,72 | -82,2 | -590,11 |
| **15** | -46,21 | -1681,8 | -94,29 | -929,05 |
| **16** | -459,76 | -4644,14 | 1611,83 | 1579,49 |
| **17** | -14,28 | -312,31 | 16,97 | -0,65 |
| **18** | 72,38 | 100,21 | 55,58 | -100,56 |
| **19** | 6,34 | -2,26 | 143,07 | -84,82 |
| **20** | -221,44 | 1305,5 | 196,22 | 5313,59 |
| **Mean** | **-19,72** | **-530,53** | **-219,78** | **40,05** |
| **SD** | **136,81** | **1599,11** | **1075,76** | **1629,6** |

**Supplementary Table 3.** Mean ERF amplitudes (z-scored pseudo neural activity index, PNAI and fT/cm) and standard deviations of N100m and P200m for all ROI and all conditions. To estimate ERF amplitudes in fT/cm, correlation coefficients (Pearson's r) were calculated between PCA time series and all averaged channels. The channel with the highest correlation value was used to estimate the ERF amplitude in fT/cm.

| **Left**<br>**Region** | **OFF listen** | **SD** | **OFF vocal** | **SD** | **ON listen** | **SD** | **ON vocal** | **SD** | **Unit** |
|---|---|---|---|---|---|---|---|---|---|
| **AC** | -25,94 | 10,63 | -26,29 | 15,66 | -22,5 | 11,54 | -21,64 | 14,91 | **PNAI** |
| | -650,29 | 403,05 | -649,49 | 452,87 | -972,16 | 879,08 | -879,28 | 759,64 | **fT/cm** |
| **STG** | -22,98 | 10,6 | -20,36 | 12,19 | -24,09 | 12,42 | -19,02 | 10,98 | **PNAI** |
| | -568,93 | 359,22 | -659,7 | 740,53 | -728,53 | 571,32 | -846,4 | 798,08 | **fT/cm** |

| **Right**<br>**Region** | **OFF listen** | **SD** | **OFF vocal** | **SD** | **ON listen** | **SD** | **ON vocal** | **SD** | **Unit** |
|---|---|---|---|---|---|---|---|---|---|
| **AC** | -19,39 | 9,5 | -20,8 | 11,67 | -20,17 | 11,37 | -17,96 | 7,93 | **PNAI** |
| | -457,26 | 229,45 | -528,12 | 255,65 | -792,59 | 695,62 | -833,98 | 711,23 | **fT/cm** |
| **STG** | -19,96 | 6,56 | -17,37 | 8,44 | -19,82 | 8,62 | -14,57 | 7,51 | **PNAI** |
| | -508,75 | 249,35 | -605,11 | 525,52 | -723,42 | 510,83 | -807,86 | 732,15 | **fT/cm** |

| **Left**<br>**Region** | **OFF listen** | **SD** | **OFF vocal** | **SD** | **ON listen** | **SD** | **ON vocal** | **SD** | **Unit** |
|---|---|---|---|---|---|---|---|---|---|
| **AC** | 20,57 | 10,54 | 20,63 | 13,09 | 20,64 | 10,6 | 15,66 | 11,28 | **PNAI** |
| | 625,26 | 327,79 | 426,54 | 435,05 | 1044,26 | 1131,2 | 777,96 | 1325,65 | **fT/cm** |
| **STG** | 18,12 | 10,55 | 16,73 | 10,16 | 21,01 | 11,28 | 15,5 | 10,37 | **PNAI** |
| | 492,68 | 259,22 | -371,19 | 3038,65 | 698,32 | 431,19 | 494,02 | 1283,48 | **fT/cm** |

| **Right**<br>**Region** | **OFF listen** | **SD** | **OFF vocal** | **SD** | **ON listen** | **SD** | **ON vocal** | **SD** | **Unit** |
|---|---|---|---|---|---|---|---|---|---|
| **AC** | 17,06 | 9,09 | 12,93 | 8,65 | 17,07 | 8,58 | 11,44 | 6,43 | **PNAI** |
| | 495,74 | 290,96 | 147,8 | 283,34 | 704,66 | 918,1 | 674,73 | 1176,35 | **fT/cm** |
| **STG** | 18,08 | 8,18 | 14,02 | 6,88 | 18,61 | 8,67 | 12,14 | 7,36 | **PNAI** |
| | 443,32 | 313 | 72,1 | 575,2 | 541,71 | 536,78 | 447,18 | 901,66 | **fT/cm** |

| **Left** | **OFF listen** | **SD** | **OFF vocal** | **SD** | **ON listen** | **SD** | **ON vocal** | **SD** |
|---|---|---|---|---|---|---|---|---|
| **AC** | 0,81 | 0,13 | 0,74 | 0,16 | 0,74 | 0,22 | 0,74 | 0,16 |
| **STG** | 0,7 | 0,14 | 0,64 | 0,18 | 0,65 | 0,19 | 0,59 | 0,16 |

| **Right** | **OFF listen** | **SD** | **OFF vocal** | **SD** | **ON listen** | **SD** | **ON vocal** | **SD** |
|---|---|---|---|---|---|---|---|---|
| **AC** | 0,69 | 0,11 | 0,72 | 0,16 | 0,72 | 0,15 | 0,67 | 0,13 |
| **STG** | 0,67 | 0,12 | 0,72 | 0,12 | 0,71 | 0,13 | 0,68 | 0,11 |

**Supplementary Table 4.** Mean ERF latencies (in s) and standard deviations of N100m and P200m for all ROI and all conditions.

**Left Hemisphere N100m**

| Region | OFF listen | SD | OFF vocal | SD | ON listen | SD | ON vocal | SD |
|---|---|---|---|---|---|---|---|---|
| AC | 0,127 | 0,013 | 0,148 | 0,024 | 0,141 | 0,025 | 0,149 | 0,03 |
| STG | 0,13 | 0,018 | 0,14 | 0,025 | 0,131 | 0,017 | 0,144 | 0,026 |
| PMC | 0,146 | 0,03 | 0,154 | 0,031 | 0,138 | 0,022 | 0,155 | 0,032 |
| IPL | 0,144 | 0,034 | 0,151 | 0,033 | 0,147 | 0,034 | 0,141 | 0,033 |

**Right Hemisphere N100m**

| Region | OFF listen | SD | OFF vocal | SD | ON listen | SD | ON vocal | SD |
|---|---|---|---|---|---|---|---|---|
| AC | 0,13 | 0,021 | 0,143 | 0,03 | 0,131 | 0,022 | 0,147 | 0,029 |
| STG | 0,129 | 0,024 | 0,142 | 0,022 | 0,131 | 0,028 | 0,157 | 0,029 |
| PMC | 0,134 | 0,025 | 0,137 | 0,028 | 0,126 | 0,017 | 0,145 | 0,027 |
| IPL | 0,137 | 0,024 | 0,15 | 0,022 | 0,133 | 0,024 | 0,146 | 0,024 |

**Left Hemisphere P200m**

| Region | OFF listen | SD | OFF vocal | SD | ON listen | SD | ON vocal | SD |
|---|---|---|---|---|---|---|---|---|
| AC | 0,26 | 0,023 | 0,26 | 0,025 | 0,265 | 0,029 | 0,255 | 0,039 |
| STG | 0,252 | 0,027 | 0,259 | 0,035 | 0,259 | 0,023 | 0,263 | 0,039 |
| PMC | 0,268 | 0,026 | 0,261 | 0,033 | 0,257 | 0,026 | 0,264 | 0,037 |
| IPL | 0,249 | 0,034 | 0,267 | 0,043 | 0,259 | 0,042 | 0,255 | 0,045 |

**Right Hemisphere P200m**

| Region | OFF listen | SD | OFF vocal | SD | ON listen | SD | ON vocal | SD |
|---|---|---|---|---|---|---|---|---|
| AC | 0,266 | 0,024 | 0,272 | 0,037 | 0,256 | 0,026 | 0,273 | 0,035 |
| STG | 0,247 | 0,033 | 0,253 | 0,031 | 0,256 | 0,03 | 0,276 | 0,032 |
| PMC | 0,246 | 0,032 | 0,268 | 0,034 | 0,266 | 0,029 | 0,264 | 0,035 |
| IPL | 0,253 | 0,04 | 0,259 | 0,03 | 0,251 | 0,029 | 0,271 | 0,032 |
